# Targeting homeostatic mechanisms of endoplasmic reticulum stress to increase susceptibility of cancer cells to fenretinide-induced apoptosis: the role of stress proteins ERdj5 and ERp57

**DOI:** 10.1038/sj.bjc.6603672

**Published:** 2007-03-13

**Authors:** M Corazzari, P E Lovat, J L Armstrong, G M Fimia, D S Hill, M Birch-Machin, C P F Redfern, M Piacentini

**Affiliations:** 1 INMI-IRCCS Lazzaro Spallanzani, Rome 00149, Italy; 2 School of Clinical Laboratory Sciences, Newcastle University, Newcastle upon Tyne NE2 4HH, UK; 3 Northern Institute for Cancer Research, Newcastle University, Paul O’Gorman Building, Medical School Framlington Place, Newcastle upon Tyne, NE2 4HH, UK

**Keywords:** endoplasmic reticulum stress, apoptosis, fenretinide, neuro-ectodermal tumours

## Abstract

Endoplasmic reticulum (ER) malfunction, leading to ER stress, can be a consequence of genome instability and hypoxic tissue environments. Cancer cells survive by acquiring or enhancing survival mechanisms to counter the effects of ER stress and these homeostatic responses may be new therapeutic targets. Understanding the links between ER stress and apoptosis may be approached using drugs specifically to target ER stress responses in cancer cells. The retinoid analogue fenretinide [N-(4-hydroxyphenyl) retinamide] is a new cancer preventive and chemotherapeutic drug, that induces apoptosis of some cancer cell types via oxidative stress, accompanied by induction of an ER stress-related transcription factor, GADD153. The aim of this study was to test the hypothesis that fenretinide induces ER stress in neuroectodermal tumour cells, and to elucidate the role of ER stress responses in fenretinide-induced apoptosis. The ER stress genes ERdj5, ERp57, GRP78, calreticulin and calnexin were induced in neuroectodermal tumour cells by fenretinide. In contrast to the apoptosis-inducing chemotherapeutic drugs vincristine and temozolomide, fenretinide induced the phosphorylation of eIF2α, expression of ATF4 and splicing of XBP-1 mRNA, events that define ER stress. In these respects, fenretinide displayed properties similar to the ER stress inducer thapsigargin. ER stress responses were inhibited by antioxidant treatment. Knockdown of ERp57 or ERdj5 by RNA interference in these cells increased the apoptotic response to fenretinide. These data suggest that downregulating homeostatic ER stress responses may enhance apoptosis induced by oxidative stress-inducing drugs acting through the ER stress pathway. Therefore, ER-resident proteins such as ERdj5 and ERp57 may represent novel chemotherapeutic targets.

Cancer cells acquire survival mechanisms, such as the inactivation of the p53 gene family (Wyllie *et al*, 1999), allowing them to circumvent death as a consequence of DNA damage. Cell death can also be induced as a result of a stress-signalling pathway activated by accumulation of misfolded or mutated proteins in the endoplasmic reticulum (ER) (Rao *et al*, 2004). Endoplasmic reticulum stress and the activation of mechanisms to protect cells against the consequences of ER malfunction are increasingly being recognised as key concepts in cancer cell biology. As the accumulation of aberrant proteins in the ER can result from chromosomal rearrangements during carcinogenesis (Khan *et al*, 2004), homeostatic responses to prevent or ameliorate the risk of apoptosis from ER stress may be a significant factor allowing transformed cells to survive the consequences of genomic instability. Endoplasmic reticulum stress pathways can also be activated in tumour cells as a result of hypoxia and other environmental factors (Feldman *et al*, 2005; Koshikawa *et al*, 2006). Shifting the balance between survival and apoptosis in favour of survival as a result of enhanced (constitutive or induced) homeostatic ER responses may facilitate metastasis and drug resistance. For example, it has been shown that the induction of the unfolded protein ER stress response (UPR) can increase the resistance of multiple myeloma cells to etoposide (Gray *et al*, 2005). Although ER stress responses represent homeostatic mechanisms allowing cells to survive the consequences of perturbations in the protein folding and processing functions of the ER, it is not clear how these mechanisms interact with signalling pathways controlling apoptosis. The point at which homeostatic mechanisms cannot cope and apoptosis is induced is critical to understanding the relationships between ER stress and apoptosis, and how ER stress can be used to increase chemotherapeutic drug targeting to tumour cells.

The retinoid analogue fenretinide [*N*-(4-hydroxyphenyl) retinamide] has an increasingly important profile as a cancer preventive and chemotherapeutic drug (Malone *et al*, 2003). Unlike most retinoids, fenretinide induces apoptosis *in vitro*; the generation of reactive oxygen species (ROS) is a common feature of fenretinide-induced apoptosis in many cell types. In SH-SY5Y neuroblastoma cells, ROS generation is apparently mediated by a ceramide-ganglioside signalling pathway and leads to induction of the ER stress-associated transcription factor GADD153/CHOP (Lovat *et al*, 2002). Furthermore, fenretinide induces NF*κ*B activity in these cells and apoptosis is mediated by the NF*κ*B pathway (Campbell Hewson *et al*, 2005). As this pathway is also implicated in ER stress responses, particularly in relation to ER stress-induced apoptosis of neuronal cells (Chen and Gao, 2002), fenretinide-induced apoptosis may be accompanied by, or result from, the activation of ER stress responses. The aim of this study was to test the hypothesis that fenretinide induces ER stress, and to elucidate the role of ER stress responses in fenretinide-induced apoptosis. Although pathways of fenretinide-induced apoptosis have been relatively well characterised in neuroblastoma cells, melanoma cells also undergo apoptosis in response to fenretinide (Montaldo *et al*, 1999a) and to test the idea that neuroectodermal cells have similar response pathways, we have studied human neuroblastoma and melanoma cell lines.

## MATERIALS AND METHODS

### Cell culture and flow cytometry

Human SH-SY5Y neuroblastoma and A375 and SK-MEL110 melanoma cells were cultured in DMEM containing 4.5 g l^−1^ glucose and supplemented with 10% foetal bovine serum as described previously for SH-SY5Y cells (Lovat *et al*, 2004). Fenretinide (Janssen-Cilag Ltd, Basserdorf, Switzerland), temozolomide (OSI Pharmaceuticals), vincristine, velcade (Janssen Pharmaceutica) or thapsigargin (Sigma Chemical Co., St Louis, MO, USA) were added in ethanol (fenretinide and vincristine) or DMSO (Lovat *et al*, 2004), with an equal volume of vehicle used to treat control cells. The SH-SY5Y cells were treated with fenretinide at a concentration of 3 *μ*M, vincristine at 10 nM, velcade at 5 nM or thapsigargin at 1.5 *μ*M. The melanoma cell lines were more resistant to drug-induced apoptosis, and for these cells, fenretinide was used at final concentrations of 10 or 15 *μ*M, as specified in the results, temozolomide was used at 1 mM, velcade at 30 nM and thapsigargin at 7.5 *μ*M. In these experiments, thapsigargin was used as a reference positive control for ER stress responses and vincristine and temozolomide as negative controls. Velcade was also used in some experiments as a comparator for fenretinide responses. The concentrations of fenretinide used were within the range of IC_50_ values described previously for a sample of 10 human melanoma cell lines (Montaldo *et al*, 1999a). Vitamin C was added to cells to a final concentration of 100 *μ*M as described previously (Lovat *et al*, 2000). Flow cytometry of fixed and propidium iodide-stained cells was used to estimate the level of cell death or apoptosis, expressed as the percentage of cells which were hypodiploid (Lovat *et al*, 2000). The generation of ROS was detected by staining cells after trypsinization with 10 *μ*M 5-(and –6)-chloromethyl-2′,7′-dihydrodichlorofluorescein diacetate (CM-H_2_DCFDA) or 1 *μ*M dihydroethidine (DHE) for 20 min at 37°C in the dark and evaluated by flow cytometry as previously described (Lovat *et al*, 2000, 2002).

### Identification of genes induced by fenretinide in SH-SY5Y cells

Endoplasmic reticulum stress genes induced by fenretinide in SH-SY5Y cells treated with or without 3 *μ*M fenretinide for 6 h were identified from microarray analysis: cells were lysed using Trizol (Invitrogen Life Technologies, Carlsbad, CA, USA) and total RNA was extracted according to the manufacturer's instructions. Poly(A)+ RNA was isolated by oligo-dT latex bead chromatography (Qiagen Inc., Valencia, CA, USA). Reverse transcriptase, primed with poly(dT), was used to synthesize Cy3- and Cy5-labelled cDNA using the Cyscribe cDNA labelling kit (Amersham Biosciences UK Ltd, Amersham, UK). Labelled cDNA probes were hybridised to glass slide micro-arrays containing 24 000 genes (Stanford University, CA, USA) and detected using a Packard MicroArray scanner (Packard BioScience, Billerica, MA, USA) with Quant Array software. Experiments were performed in triplicate and expression confirmed by Western blotting and reverse transcriptase-polymerase chain reaction (RT-PCR) in separate experiments.

### Western blotting

Total protein was extracted from cell pellets and separated by electrophoresis through 12% SDS-PAGE gels (30 *μ*g per track) and blotted onto nitrocellulose as described previously (Lovat *et al*, 2004). Blots were probed with antibodies to GADD153 (Santa Cruz Biotechnology Inc., Santa Cruz, CA, USA, diluted 1 : 1000), ERp57 (Stressgen, Victoria, BC, Canada, diluted 1 : 5000, and Santa Cruz, sc-23886, diluted 1 : 100 000), ERdj5 (Abnova Corporation, Taipei, Taiwan; DNAJC10 polyclonal antibody H00054431-A01, diluted 1 : 2000), calreticullin (Stressgen, diluted 1 : 2000), calnexin (Santa Cruz, diluted 1 : 1000), GRP78 (Santa Cruz, diluted 1 : 1000), eIF2*α* (Cell Signaling, diluted 1 : 1000), phosphorylated eIF2*α* (P-eIF2*α*; Cell Signaling, diluted 1:1000) or cleaved caspase-3 (Cell Signalling Technology, Danvers, MA, USA, diluted 1:1000). As a loading control, blots were probed with *β*-tubulin (Sigma, Poole, UK) diluted 1 : 5000 (Lovat *et al*, 2002) or *β*-actin (Sigma, Poole, UK, diluted 1 : 5000). For detection, blots were incubated with secondary peroxidase-conjugated antibodies (Jackson ImmunoResearch Inc., West Grove, PA, USA, diluted 1 : 5000, or Upstate Biotechnology, UK, diluted 1 : 2000) and visualized using the ECL Plus system (Amersham Biosciences, UK) (Corazzari *et al*, 2003).

### Reverse transcriptase-polymerase chain reaction

For analysis of XBP-1 splicing, the human XBP-I sequence was amplified by RT–PCR with the primer pair AAACAGAGTAGCAGCTCAGACTGC and CCTTCTGGGTAGACCTCTGGGAG (Calfon and Harding, 2004). RT–PCR was used to quantify ERdj5 and GAPDH (as a loading control) in RNA extracted from cells treated with thapsigargin or fenretinide. For these PCR analyses, cells were lysed and total RNA was extracted with Trizol as above. A poly(dT) primer (1 mM) was used to generate cDNA from 2 *μ*g of RNA. The human ERdj5 sequence was amplified with the primer pair GCCATTTTAGTGGGCACAGATCAGG and CAGCCAGCCAATACCAGCAGCA and the human ERp57 sequence was amplified with the primer pair ACGTGCTAGAACTCACGGA and ACTGAAGCTGGTCCTGCCTG. Amplification of human GAPDH sequence was with the primer pairs GATATCGCCGCGCTCGTCGTCG and AGGTAGTCAGTCAGGTCCCGGC. The number of cycles used for each primer pair in the PCR reactions was adjusted so that amplification remained within an approximately linear range.

### Quantification of mRNA by real-time RT–PCR

Total RNA was isolated from cell pellets using the RNeasy Mini kit (Qiagen, Crawley, UK). RNA was reverse-transcribed using Promega's Reverse Transcription System according to the manufacturer's instructions, using random hexamer primers. For human ERdj5 and ERp57, real-time PCR was performed on 20–40 ng cDNA using predesigned TaqMan Gene Expression Assays, in combination with the TaqMan Universal PCR master mix (Applied Biosystems, Warrington, UK). Appropriate controls for nonspecific amplification and contamination were included. A GeneAmp 5700 Sequence Detection System (Applied Biosystems) was used for real-time PCR amplification. As internal standard, *β*-actin was measured simultaneously using the endogenous control assay provided by Applied Biosystems. Polymerase chian reaction amplification procedures followed the manufacturer's instructions. Briefly, the thermocycling program consisted of one cycle at 50°C for 2 min followed by 95°C for 10 min and 40 cycles at 95°C (15 s) and 60°C (1 min). The data were analyzed using the GeneAmp Sequence Detection System software. For ATF4 mRNA and mRNA for the ribosomal protein L34 used as an internal control, primers were designed using Primer Express 1.0 Software (Applied Biosystems) and amplification was performed using a LightCycler with DNA Master SYBR Green I kit (Roche Applied Science, Monza, Italy) as specified by the manufacturer'sinstructions. Primers for ATF4 were GTGGCCAAGCACTTCAAACC and CCCGGAGAAGGCATCCTC, and for ribosomal protein L34: GTCCCGAACCCCTGGTAATAGA and GGCCCTGCTGACATGTTTCTT. The comparative C_t_ method (ΔΔC_t_) was used for relative quantification of gene expression.

### siRNA-mediated knockdown

For both ERdj5 and ERp57, two different siRNAs were evaluated against a scrambled siRNA control, and at doses of 20 and 40 nM for each siRNA in all cell lines. The siRNA knockdown experiments were performed by plating 0.25 × 10^6^ cells in six-well plates overnight and transfecting for 18 h with 20 or 40 nM (final concentrations) siRNA using lipofectamine in Optimem culture medium (Invitrogen) (Lovat *et al*, 2004). After incubation overnight at 37°C, fetal bovine serum was added to 10% and cells incubated with or without 3 or 10 *μ*M fenretinide for a further 24 h. Replicates were used for analysis of apoptosis and to verify knockdown by real-time PCR and Western blotting. siRNAs, supplied desalted and annealed by Eurogentec (Southampton, UK) were: ERdj5-2 GGAGGAGAUUGUUUGACUU; ERdj5-3 AGACAAGCUUUCAAGAAAU; ERp57-1 GGACAAGACUGUGGCAUAU; ERp57-2 GAAGCUAAAUCCAAAGAAA and scrambled nonsilencing control UUCUCCGAACGUGUCACGU.

### Data analysis

Statistical analysis of apoptosis and gene induction in each cell line was by one-way ANOVA with all pairwise comparisons after Bonferroni correction. Analysis of gene knockdown experiments with respect to apoptosis or gene expression as response variables was by a General Linear Model (GLM) with cell line, fenretinide treatment and siRNA type as independent factors, and siRNA dose as a linear effect (linear contrast). Analysis of apoptosis in response to different ER stress inducers or cytotoxic drugs after knockdown with control, ERdj5 or ERp57 siRNA was by two-way ANOVA (drug and siRNA type) with Bonferroni-corrected hypothesis tests for specific comparisons. Analyses were carried out using Systat version 10 (Systat Software UK Ltd, London, UK). Error bars on graphs are 95% confidence intervals, except where the mean and range of duplicate measurements are shown, as explained in the legends.

## RESULTS

### Activation of ER stress responses by fenretinide

**Figure 1:**
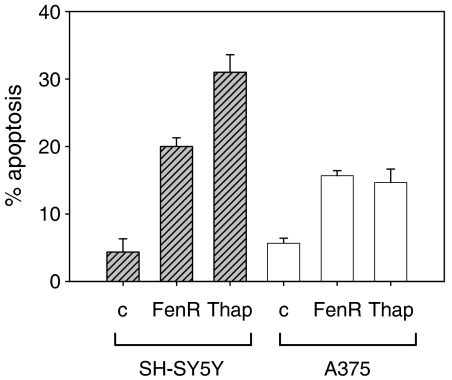
Fenretinide or thapsigargin induced cell death in SH-SY5Y and A375 cells. Cells were treated with fenretinide at 3 *μ*M (SH-SY5Y cells) or 15 *μ*M (A375 cells), or thapsigargin at 1.5 *μ*M (SH-SY5Y cells) or 7.5 *μ*M (A375 cells) for 24 h and cell death (apoptosis) measured by flow cytometry. Bar heights are mean percentage apoptosis of three replicates+95% confidence interval (error bars).

To test the hypothesis that fenretinide activates ER stress responses, a 24K microarray was screened for the induction of ER stress genes in SH-SY5Y neuroblastoma cells after a 6-h treatment with fenretinide. In addition to GADD153, four other genes associated with ER stress were induced greater than twofold: ERdj5 (an ER-resident protein containing DnaJ and thioredoxin domains; Cunnea *et al*, 2003), ERp57 (GRP58; an ER-resident protein-disulphide isomerise; Frickel *et al*, 2004), calreticulin and calnexin (both ER-resident chaperones; Bedard *et al*, 2005). To verify the induction of these genes in independent experiments and to assess their relevance as markers of fenretinide response in neuroectodermal cells, we studied the activation of these genes in SH-SY5Y neuroblastoma cells and A375 melanoma cells (Kozlowski *et al*, 1984). We have also studied SK-Mel-110 melanoma cells (Albino *et al*, 2000), but as the results for these cells were, in all respects, similar to A375 cells, only data for the A375 cells are reported here. In addition, as a positive control, we compared the fenretinide response of these cells with their response to thapsigargin, a well-characterised inducer of ER stress (Kass and Orrenius, 1999). Both agents induced apoptosis in these cell lines (Figure 1); however, as has been reported for other melanoma lines with respect to fenretinide sensitivity (Montaldo *et al*, 1999), the melanoma cells were more resistant than SH-SY5Y cells and needed three- to five-fold higher concentrations of either agent to induce comparable levels of cell death. The time course of fenretinide-induced apoptosis has been published previously (Lovat *et al*, 2000); in the experiments reported here, we have treated cells with fenretinide or other agents to achieve an apoptotic index of 20–25% to avoid early molecular events in stress responses being masked by high levels of cell death.

**Figure 2:**
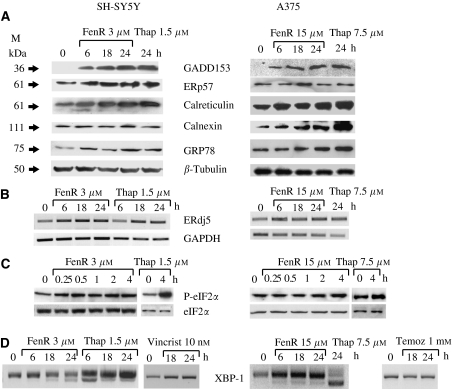
The induction of ER-stress genes in response to fenretinide or thapsigargin in SH-SY5Y and A375 cells. (**A**) Western blots of total protein extracted from SH-SY5Y cells (left-hand column) or A375 cells (right-hand column) treated with thapsigargin for 24 h (Thap; 1.5 *μ*M for SH-SY5Y cells or 7.5 *μ*M for A375 cells) or fenretinide (FenR; 3 *μ*M for SH-SY5Y cells or 15 *μ*M for A375 cells) for 0, 6, 18 or 24 h. Blots were probed with antibodies to GADD153, ERp57, calreticulin, calnexin, GRP78 and, as a loading control, *β*-tubulin. Apparent molecular weights of the relevant bands are given on the left. (**B**) Reverse transcription–polymerase chain reaction was used to quantify ERdj5 and GAPDH (as a loading control) in RNA extracted from cells treated with thapsigargin or fenretinide. In the PCR reactions the number of cycles used for each primer pair was adjusted so that amplification remained within an approximately linear range. (**C**) Western blots (as in A) probed using antibodies for eIF2*α* and phosphorylated eIF2α (P-eIF2*α*). Cells were treated with fenretinide for 0.25, 0.5, 1, 2 or 4 h, or thapsigargin for 4 h, (**D**) induction of XBP-1 splicing in response to fenretinide or thapsigargin as in (**B)** with additional experiments to show lack of XBP-1 splicing in cells treated with 10 nM vincristine (SH-SY5Y cells) or 1 mM temozolomide (A375 cells) for up to 24 h.

Western blots confirmed the induction of GADD153, ERp57, calreticulin and calnexin in response to fenretinide or thapsigargin (Figure 2A). In these experiments, the Western blots were also probed for GRP78 (Gene Symbol HSPA5, also known as BiP), an additional ER stress marker (Bertolotti *et al*, 2000): this was also induced by fenretinide and thapsigargin (Figure 2A). We initially used RT–PCR to confirm the induction of ERdj5 mRNA under the same conditions (Figure 2B). However, subsequent experiments using real-time quantitative PCR confirmed the induction of ERdj5 and also ERp57 mRNA (see below).

**Figure 3:**
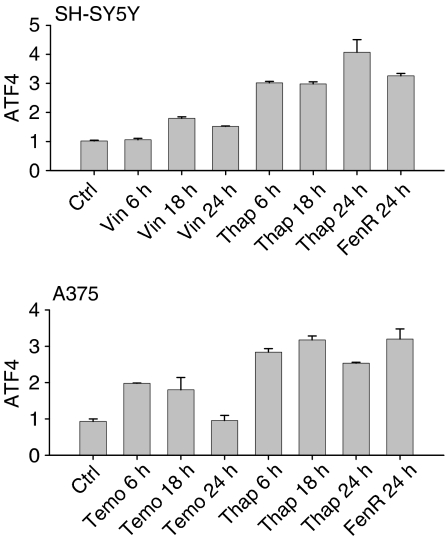
Induction of ATF4 mRNA in SH-SY5Y cells (upper graph) and A375 cells (lower graph) in response to treatment with fenretinide (3 or 15 *μ*M respectively) for 24 h, or with thapsigargin (1.5 *μ*M or 7.5 *μ*M, respectively) or 10 nM vincristine (SH-SY5Y cells) or 1 mM temozolomide (A375 cells) for 6, 18 and 24 h. Bar heights and error bars are means and upper range of duplicate samples or means and upper standard deviation of triplicate samples (thapsigargin data) relative to the control (vehicle) treatment.

The induction of eIF2*α* phosphorylation and splicing of XBP-1 mRNA are events used to define ER stress (Brostrom and Brostrom, 1998; Calfon *et al*, 2002). Phosphorylation of eIF2*α* was induced by thapsigargin (1.5 *μ*M for SH-SY5Y cells, 7.5 *μ*M for melanoma cells) within 15 min of treatment with fenretinide in SH-SY5Y cells and within 4 h of treatment in A375 cells (Figure 2C). Under similar conditions, the splicing of XBP-1 mRNA was induced within 6 h of thapsigargin treatment and within 6–18 h after treatment with fenretinide (Figure 2D). To ask if ER stress responses were a general response of these cells to cytotoxic agents, the cells were treated for up to 24 h with vincristine (SH-SY5Y cells) or temozolomide (A375 cells), cytotoxic agents used clinically to treat neuroblastoma or melanoma, respectively. In contrast to fenretinide or thapsigargin, these agents did not induce detectable XBP-1 splicing up to 24 h after treatment (Figure 2D). Increased expression of the transcription factor ATF4 is also a characteristic of ER stress responses. Fenretinide induced a three-fold increase in ATF4 expression after 24 h. Although slight induction was seen in SH-SY5Y cells treated with vincristine for 18 h, and in A375 cells treated with temozolomide for 6 h, this did not reach the levels induced in response to fenretinide (Figure 3). These data indicate that fenretinide induces ER stress in SH-SY5Y and A375 neuroectodermal tumour cells.

### Blocking ROS inhibits the fenretinide-induced ER-stress response

**Figure 4:**
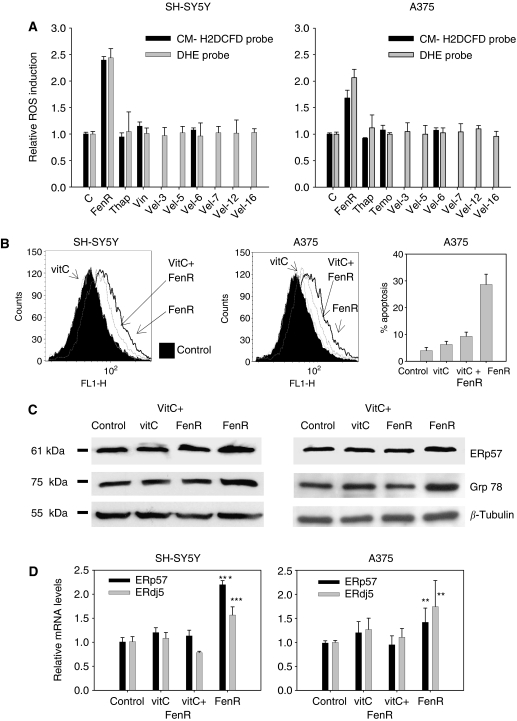
Blocking ROS with an antioxidant inhibits fenretinide-induced stress responses in SH-SY5Y and A375 cells. In all experiments, fenretinide was used at 3 *μ*M to treat SH-SY5Y cells and at 10 *μ*M to treat A375 cells; vitamin C (100 *μ*M) was added to cells 2 h before adding fenretinide and incubation continued in the presence of both reagents for a further 22 h. (**A**) A comparison of ROS induction in response to fenretinide (FenR), thapsigargin (Thap), vincristine (Vin; SH-SY5Y cells), temozolomide (Temo; A375 cells) or velcade (Vel)in SH-SY5Y cells (left panel) and A375 cells (right panel). ROS levels are expressed relative to control vehicle treatment and were measured with two different probes for reactive oxygen intermediates: CM-H_2_DCFDA or DHE in separate experiments. Bar heights are means plus upper 95% confidence interval of CM- H_2_DCFDA fluorescence (black bars) or DHE fluorescence (grey bars) (*n*=3, but *n*=6 for control and fenretinide-treated cells). ROS was measured after 6 h for control, fenretinide, thapsigargin, vincristine or temozolomide treatments, but for velcade after 3, 5, 7, 12 and 16 h using the DHE probe and 6 h with CM-H_2_DCFDA or DHE probes. (**B**) left and centre panels: flow cytometry CM- H_2_DCFDA-fluorescence profiles for SH-SY5Y (included as a positive control) or A375 cells showing the induction of ROS by fenretinide (FenR) and the reduction in fenretinide-induced ROS in cells after pretreatment with 100 *μ*M vitamin C 2 h before adding fenretinide (VitC+FenR). Vitamin C alone (VitC) did not increase ROS above the background control. Ordinate, event counts; abscissa, fluorescence signal intensity (FL1-H). Right panel: abrogation of fenretinide-induced apoptosis in A375 cells by vitamin C (mean and upper 95% confidence interval of three replicates). (**C**) Western blots showing that the fenretinide-induced increase in expression of ERp57 and GRP78 protein in response to fenretinide was inhibited by pretreatment of SH-SY5Y or A375 cells with vitamin C. (**D**) The fenretinide-induced increase in ERp57 and ERdj5 mRNA in SH-SY5Y and A375 cells, as measured by real-time quantitative PCR, was inhibited by pretreatment with vitamin C. Bar heights are means plus 95% confidence limit for triplicates samples. Contrasts were used within one-way ANOVA to compare relative ERp57 or ERdj5 mRNA levels in cells treated with fenretinide or fenretinide plus vitamin C; ^**^P<0.01, ^***^P<0.001. For ERp57 or ERdj5 in SH-SY5Y cells, one-way ANOVA F_3,8_>39, *P*<0.0001, contrasts: fenretinide versus fenretinide plus vitamin C F_1,8_>112, *P*<0.0001. For ERp57 or ERdj5 in A375 cells, one-way ANOVA F_3,12_>6, *P*⩽0.007, contrasts: fenretinide versus fenretinide plus vitamin C F_1,12_>13, *P*=0.003. In A-D, control, vehicle control; vitC, cells treated with 100 *μ*M alone; FenR, SH-SY5Y or A375 cells treated with 3 *μ*M or 15 *μ*M fenretinide, respectively; vitC+FenR, cells pretreated with 100 *μ*M vitamin C 2 h before adding fenretinide.

Previous studies have shown that antioxidants block ROS induction and apoptosis in response to fenretinide in SH-SY5Y cells (Lovat *et al*, 2000, 2002). In SH-SY5Y cells, ROS production reaches a maximum after about 6 h of fenretinide treatment (Lovat *et al*, 2000). Fenretinide also induces ROS in A375 cells (Figure 4A). In contrast, ROS was not induced by thapsigargin in SH-SY5Y or A375 cells (Figure 4A), or by vincristine in SH-SY5Y cells or temozolomide in A375 cells. Therefore, increased ROS is not an early event in response to ER stress induced by thapsigargin, and is not a general response to cytotoxic agents. Recently, it has been shown that a new anticancer drug, the proteasome inhibitor velcade (bortezomib, PS-341), induces ER-stress responses in some cell types (Fribley *et al*, 2004; Obeng *et al*, 2006) and which is apparently mediated by ROS induction in mantle-cell lymphoma cells (Perez-Galan *et al*, 2006). However, this drug was also ineffective at inducing ROS in SH-SY5Y or A375 cells after 3–16 h of treatment.

The antioxidant vitamin C blocked the induction of ROS in A375 cells in response to fenretinide (Figure 4B, centre panel) and abrogated fenretinide-induced apoptosis (Figure 4B, right panel). In SH-SY5Y and A375 cells, vitamin C blocked the fenretinide-induced increase in GRP78 and ERp57 protein and mRNA for ERp57 and ERdj5 (Figure 4C and D). In these experiments, the measurements of ERdj5 and ERp57 mRNA levels by real-time quantitative PCR show that fenretinide induced a 1.5-2-fold increase in mRNA levels for these two genes. This is additional verification of the RT–PCR experiments for ERdj5, and also shows that the induction of ERp57 protein is a result of increased mRNA levels. These data suggest that fenretinide induces ER stress in these cells via the induction of ROS.

### Increasing fenretinide-induced cell death by knockdown of ER-stress-response proteins

**Figure 5:**
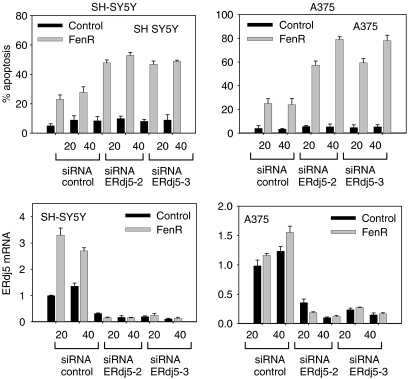
SiRNA-mediated knockdown of ERdj5 increased apoptosis in response to fenretinide in SH-SY5Y and A375 cells. In all experiments, SH-SY5Y cells were treated with fenretinide at 3 *μ*M and A375 cells with fenretinide at 10 *μ*M. For the A375 cells, a slightly lower concentration of fenretinide was used to ensure that any increased cell death remained within a 20–80% range. Cell death (apoptosis) was measured by flow cytometry. Two different ERdj5 siRNAs were evaluated against a scrambled siRNA control, and at doses of 20 and 40 nM. The upper graphs show the percentage apoptosis of SH-SY5Y cells (left-hand column) and A375 cells (right-hand column) and the lower graphs show real-time quantitative PCR data to verify ERdj5 knockdown for SH-SY5Y cells and A375 cells, respectively. Cells were transfected with 20 or 40 nM scrambled control siRNA (siRNA control), 20 or 40 nM ERdj5-2 siRNA, or 20 nM or 40 nM ERdj5-3 siRNA, and subsequently treated with (gray bars) or without (black bars) fenretinide. Real-time quantitative PCR data are expressed relative to the 20 nM scrambled siRNA control in the absence of fenretinide. On all graphs, error bars are the upper 95% confidence limits.

**Figure 6:**
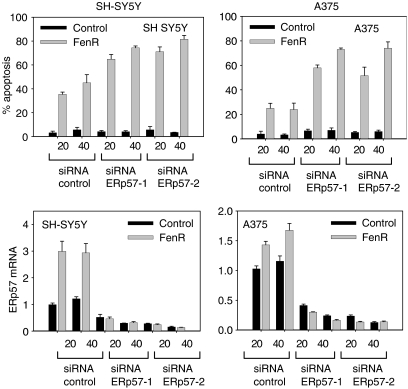
SiRNA-mediated knockdown of ERp57 increased apoptosis in response to fenretinide in SH-SY5Y and A375 cells. Experimental details as in the legend to Figure 6. Cells were transfected with 20 or 40 nM scrambled control siRNA (siRNA control), 20 or 40 nM ERp57-1 siRNA, or 20 or 40 nM ERp57-2 siRNA, and subsequently treated with (gray bars) or without (black bars) fenretinide. Real-time quantitative PCR results to verify ERp57 knockdown are shown in the lower graphs. The reduction in expression of ERp57 was also evident on Western blots (data not shown) and shown in independent experiments (Figure 7).

Since the induction of ER stress proteins may represent a homeostatic response to protect the cell from environmental stress, we predicted that the apoptotic response to fenretinide would be increased by knockdown of ER stress response proteins. To test this hypothesis with respect to the ER-stress chaperones ERp57 and ERdj5, SH-SY5Y and A375 cells were treated with fenretinide after siRNA-mediated knockdown of expression of ERdj5 or ERp57. Real-time quantitative PCR of mRNA extracted from parallel samples at 24 h confirmed that the fenretinide-induced increase in ERp57 and ERdj5 mRNA levels was significantly blocked by siRNAs for ERp57 and ERdj5, respectively, but not by the control siRNA (Figures 5 and 6; Table 1). There was no difference in efficacy between the two different siRNA sequences used for each target mRNA, and no differences between the results for the two doses (20 and 40 nM) used (Table 1). Transfection with these siRNAs and without subsequent fenretinide treatment also reduced ERdj5 (Figure 5) or ERp57 mRNA levels (Figure 6) to half or less of those in untreated control cells transfected with the scrambled siRNA.

Under these conditions of ERp57 or ERdj5 knockdown, there was a two-fold increase in cell death in response to fenretinide compared to the scrambled siRNA control (Table 1; Figures 5 and 6). As with ERdj5 and ERp57 mRNA levels, there was no difference in response to the two different target siRNAs used for each of ERp57 or ERdj5. However, in contrast to the effects of siRNA knockdown on ERdj5 and ERp57 transcript levels, there was a significant siRNA dose effect on cell death (0.05>*P*>0.01; Table 1) with the 40 nM siRNA concentration producing greater apoptosis in combination with fenretinide compared to the 20 nM concentration (Figures 5 and 6). Under these conditions of ERp57 or ERdj5 knockdown, there was a two-fold increase in cell death in response to fenretinide compared to the scrambled siRNA control (Figures 5 and 6).

**Figure 7:**
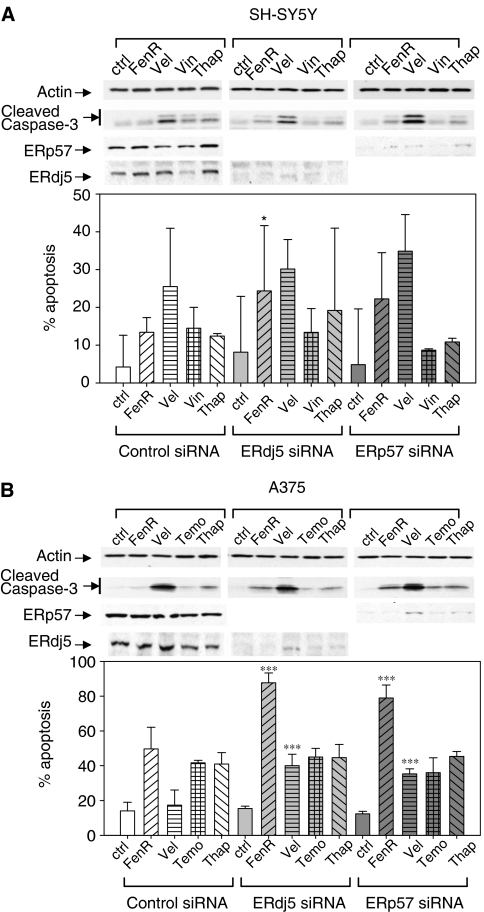
Apoptosis in SH-SY5Y cells (**A**) and A375 cells (**B**) after gene knockdown with control, ERdj5-2 or ERp57-3 siRNA. Apoptosis in response to control vehicle, fenretinide (3 or 10 *μ*M), velcade (5 or 30 nM), vincristine (10 nM) or temozolomide (1 mM) (SH-SY5Y or A375 cells, respectively), or thapsigargin (1.5 *μ*M for SH-SY5Y or 7.5 *μ*M for A375 cells) was measured quantitatively by flow cytometry of PI-stained cells; the detection of cleaved caspase-3 on Western blots (a technique that is less sensitive than flow cytometry), relative to *β*-actin as a loading control, was used as additional verification of apoptosis (upper panels in A and B). Confirmation of ERp57 and ERdj5 protein knockdown was assessed by Western blotting. Bar heights indicate the mean with error bars showing the upper 95% confidence limits. Statistical comparisons were hypothesis tests (Bonferroni-corrected) from within a two-way ANOVA in which the effect of siRNA knockdown of ERdj5 or ERp57 was compared with the scrambled control for that drug treatment; ^*^P<0.05; ^***^P<0.0001.

To ask if knockdown of ERdj5 or ERp57 enhanced the response to apoptosis induced by other cytotoxic agents, cells were exposed to fenretinide, velcade, vincristine (SH-SY5Y cells), temozolomide (A375 cells) or thapsigargin after transfection with the scrambled siRNA control, ERdj5-3 or ERp57-2 siRNA. Apoptosis was assessed quantitatively by flow cytometry of PI-stained cells; as a separate check on interpretation of flow cytometry data, apoptosis was assessed qualitatively by the appearance of cleaved caspase-3 on Western blots although this is a less sensitive technique (Figure 7). To extend qPCR data on the efficiency of mRNA knockdown (Figures 5 and 6), reduction of ERp57 or ERdj5 protein by ERp57 or ERdj5 siRNA, respectively, was confirmed by Western blotting (Figure 7). For SH-SY5Y cells, knockdown of ERdj5 significantly increased apoptosis in response to fenretinide (F_1,30_=8.6, Bonferroni-corrected *P*<0.05, 8 comparisons), although in this experiment the effect of ERp57 knockdown was not statistically significant (*P*=0.192; Figure 7). All other drug treatments were not enhanced significantly by knockdown of ERdj5 or ERp57. In A375 cells, apoptosis in response to fenretinide or velcade was significantly enhanced by knockdown of ERdj5 or ERp57 (F_1,30_>73, *P*<0.0001). However, knockdown of ERdj5 or ERp57 did not significantly enhance apoptosis in response to temozolomide or thapsigargin (F_1,30_<7.3, Bonferroni-corrected *P*>0.05). These results show that knockdown of ER stress response proteins ERdj5 or ERp57 enhances apoptotic responses to some ER stress inducing agents, although there may be cell specificity in enhancement with different ER stress inducers.

## DISCUSSION

These results show that fenretinide induces characteristics of an ER stress response in these neuroectodermal tumour cell lines. The observation that the antioxidant vitamin C inhibited the induction of ER stress-response genes and inhibited the induction of ROS in response to fenretinide, suggests that the ER stress response is a consequence of ROS induced by fenretinide. Downregulating two of these stress response genes, ERdj5 and ERp57, increased the amount of cell death in response to fenretinide and this suggests that these genes were induced as part of a homeostatic response to alleviate stresses imposed by fenretinide treatment. The nature of the fenretinide-induced ER stress is not known, but might result from lipid peroxide free radicals as a result of 12-lipoxygenase activity (Lovat *et al*, 2004). Since both GADD153 and NFκB are induced in SH-SY5Y cells by fenretinide (Lovat *et al*, 2003; Campbell Hewson *et al*, 2005), the ER stress may result from both UPR and protein overload, since evidence from other cell types suggests that these can be mediated by distinct signalling pathways (Pahl, 1999). A fundamental question raised by these studies is whether fenretinide simultaneously activates distinct proapoptotic and prosurvival (protection) mechanisms with apoptosis resulting if the balance is tipped in favour of the proapoptotic pathway. Alternatively, there may be a temporal separation of protective mechanisms and proapoptotic signalling, the latter being initiated as the capacity to protect against ROS-induced damage is exceeded.

Many ER stress-induced proteins share similarities in structure or function. ERp57 is a thiol-oxidoreductase chaperone of the protein disulphide isomerase (PDI) family and can be physically associated with calnexin and calreticulin (Bedard *et al*, 2005). ERdj5 is less well characterized, but in addition to the DnaJ and thioredoxin domains it is a chaperone that co-localises with PDIs, has a PDI-like domain and interacts with GRP78 (Cunnea *et al*, 2003). Thus, the accumulation of unfolded or misfolded proteins within the ER is likely to result in the induction of proteins to facilitate an increase in the rate at which such proteins can be correctly folded and exported. Recent studies have shown that downregulation of GRP78 increases hepatoma cell toxicity to troglitazone (Maniratanachote *et al*, 2005), and downregulation of ERp57 significantly enhances the neurotoxicity of prions, an ER stress inducer of neuronal cells (Castilla *et al*, 2004). Clearly, downregulating components of homeostatic stress responses, or interfering with the functions of these proteins, may lead to new approaches for enhancing the efficacy of drugs that induce apoptosis of tumour cells via ER stress (Linder and Shoshan, 2005).

The observation that knockdown of ERdj5 or ERp57 enhanced the extent of cell death induced by fenretinide or velcade adds considerable support to the view that downregulating ER stress responses may be therapeutically valuable; the ER resident proteins ERdj5 and ERp57 may thus be targets for the development of novel chemotherapeutic strategies. The down-regulation of ER stress responses may only potentiate cell death induced by certain classes of therapeutic drugs since the response to DNA-damaging agents vincristine and temozolomide was not affected by siRNA-mediated knockdown of ERdj5 or ERp57. These drugs did not induce responses characteristic of ER stress. Velcade, a drug currently in clinical trials for haematological malignancies (particularly multiple myeloma), non-small-cell lung carcinoma and malignant melanoma (Spano *et al*, 2005), induces ER stress possibly as a result of a failure of the 26S proteasome system to clear unwanted proteins from the ER compartment (Fribley *et al*, 2004; Nawrocki *et al*, 2005; Fribley and Wang, 2006; Obeng *et al*, 2006). Although knockdown of ERdj5 or ERp57 increased cell death in response to fenretinide or velcade, these drugs may induce ER stress via different stress sensors since velcade, unlike fenretinide, did not induce ROS in SH-SY5Y or A375 cells. Further studies are needed to elucidate the link between fenretinide-induced ROS and UPR or protein-overload mechanisms.

Although ER stress was induced by thapsigargin, cell death in response to this agent was not affected by knockdown of ERdj5 or ERp57. Like velcade, thapsigargin did not induce ROS or oxidative stress in SH-SY5Y cells or A375 cells, and similar results have been reported for the effect of thapsigargin on leukaemia cells (Zhang *et al*, 2006). Thapsigargin disrupts Ca^2+^ homeostasis by inhibiting an ER Ca^2+^-ATPase; although ER stress is a consequence, recent studies for SH-SY5Y cells and other cell types suggest that thapsigargin induces cell death by activation of separate ER- and mitochondria-dependent pathways (Kitamura *et al*, 2003; Zhang and Armstrong, 2006). Therefore, the involvement of two separate death signalling pathways in response to thapsigargin may reduce sensitivity to down-regulation of ER-stress chaperones, particularly if a mitochondria-dependent mechanism is a major driver of cell death in response to thapsigargin.

Many existing chemotherapeutic drugs induce DNA or chromosomal damage, leading to cell death via the intrinsic mitochondrial pathway culminating in the release of cytochrome *c* (Kaufmann and Earnshaw, 2000), or by the activation of cell-surface death receptors. These drugs are relatively indiscriminate, relying for their success on targeting proliferating cells rather than tumour cells specifically. In contrast, fenretinide and velcade appear to be relatively specific for tumour cells (O’Donnell *et al*, 2002; Fernandez *et al*, 2005), and we speculate that there may be a link between this tumour-cell specificity and their ability to induce ER stress. Clearly, the use of drugs that induce cell death predominantly via ER-stress, such as fenretinide or velcade, in combination with agents to reduce homeostatic responses to ER stress, has the potential to facilitate greater tumour-cell targeting than is currently possible with conventional chemotherapeutic drugs.

## Figures and Tables

**Table 1:** Analysis of siRNA knockdown with respect to apoptosis and levels of ERdj5 and ERp57 mRNA levels in three cell lines SH-SY5Y, SK-Mel-110 and A375

**Apoptosis:**	**ERdj5**		**ERp57**	
**Effect**	**F**	***P***	**F**	***P***
Fenretinide (F_1,101_)	320	<0.0001	647	<0.0001
SiRNA (F_2,101_)	23.8	<0.0001	40.2	<0.0001
siRNA dose (F_1,101_)	4.29	0.041	5.5	0.02
				
Hypothesis tests				
Difference between target siRNAs	0.07	0.79	0.107	0.74
Difference between target siRNA and control siRNA	>34	<0.0001	>57	<0.0001
				
**mRNA levels**				
Effect	F	*P*	F	*P*
Fenretinide (F_1,101_)	15.6	<0.001	15.7	<0.001
SiRNA (F_2,101_)	113.2	<0.0001	109.8	<0.0001
siRNA dose (F_1,101_)	0.79	0.38	1.66	0.2
Hypothesis tests:				
Difference between target siRNAs	0.03	0.87	1.99	0.16
Difference between target siRNA and control siRNA	>167	<0.0001	>145	<0.0001

Analysis was done using a General Linear Model (GLM) with, as factors, cell line, fenretinide treatment (no fenretinide or single dose as specified in the legends to Figure 5 and 6) and siRNA type (scrambled control and two different siRNA for both ERdj5 and ERp57); siRNA dose (20 or 40 nM) was included as a linear effect. Separate analyses were performed for ERp57 and ERdj5 with respect to apoptosis and mRNA levels. The table gives the value of the F statistic (degrees of freedom are given for each main effect) and the associated probability (*P*) for the ERp57 and Erdj5 GLMs. Differences between cell lines in the context of the analysis are trivial and are not reported in the table below.
